# 2D:4D Suggests a Role of Prenatal Testosterone in Gender Dysphoria

**DOI:** 10.1007/s10508-020-01630-0

**Published:** 2020-01-23

**Authors:** Mostafa Sadr, Behzad S. Khorashad, Ali Talaei, Nasrin Fazeli, Johannes Hönekopp

**Affiliations:** 1 Transgender Studies Centre, Psychiatry and Behavioral Sciences Research Centre, Mashhad University of Medical Sciences, Mashhad, Iran; 2 Department of Psychology, Northumbria University, Newcastle upon Tyne, NE1 8ST UK

**Keywords:** Gender dysphoria, Transsexualism, Prenatal testosterone, Digit ratio 2D:4D, Meta-analysis

## Abstract

Gender dysphoria (GD) reflects distress caused by incongruence between one’s experienced gender identity and one’s natal (assigned) gender. Previous studies suggest that high levels of prenatal testosterone (T) in natal females and low levels in natal males might contribute to GD. Here, we investigated if the 2D:4D digit ratio, a biomarker of prenatal T effects, is related to GD. We first report results from a large Iranian sample, comparing 2D:4D in 104 transwomen and 89 transmen against controls of the same natal sex. We found significantly lower (less masculine) 2D:4D in transwomen compared to control men. We then conducted random-effects meta-analyses of relevant studies including our own (*k* = 6, *N* = 925 for transwomen and *k* = 6, *N* = 757 for transmen). In line with the hypothesized prenatal T effects, transwomen showed significantly feminized 2D:4D (*d* ≈ 0.24). Conversely, transmen showed masculinized 2D:4D (*d* ≈ − 0.28); however, large unaccounted heterogeneity across studies emerged, which makes this effect less meaningful. These findings support the idea that high levels of prenatal T in natal females and low levels in natal males play a part in the etiology of GD. As we discuss, this adds to the evidence demonstrating the convergent validity of 2D:4D as a marker of prenatal T effects.

## Introduction

Gender dysphoria (GD) describes a distressing mismatch between one’s gender identity and natal sex (American Psychiatric Association, 2013). Not much is known about the origin of GD (Meyer-Bahlburg, 2013), but twin studies show that genetic factors play a substantial role (for a review, see Polderman et al., 2018). Also, prenatal testosterone (T) might have an effect on GD.

### A Role for Organizational Testosterone Effects

A vast body of experimental research suggests that androgen action during critical periods in early development plays a crucial role in the creation of somatic and behavioral sex differences across mammalian species (for reviews, see McCarthy & Arnold, 2011; Motta-Mena & Puts, 2017). Behavioral effects on copulation, aggression, and rough-and-tumble play result from organizational (i.e., permanent) effects of androgens or their metabolites on the brain. A number of key principles have been distilled from animal experiments: (1) behaviors that are affected by organizational androgen effects show sex differences; (2) critical periods for organizational androgen effects are marked by higher T production in males than females; (3) organizational androgen effects contribute to between- as well as within-sex differences; (4) finally, within the physiological range, organizational androgen effects are roughly linear (Hines, Constantinescu, & Spencer, 2015).

In early human development, T production is higher in males than in females, particularly from about weeks 12 to 16 during gestation (Reyes, Winter, & Faiman, 1973) and from 1 to 5 months after birth (Lamminmäki et al., 2012), which suggests that these perinatal periods are critical for organizational T effects. Later in life, puberty might be a final period for organizational effects (see Schulz, Molenda-Figueira, & Sisk, 2009). In humans, experiments into such effects are not feasible, but observations on individuals who experienced atypical hormonal effects during development (e.g., due to medical conditions) suggest prenatal T effects on a suite of psychological variables (Berenbaum & Beltz, 2016). Among them are a moderate effect of prenatal T on the gender of desired romantic partners (e.g., Khorashad et al., 2016, 2017; Zucker et al., 1996) and a strong effect on human play behavior (Hines, 2010), which in itself shows very large sex differences regarding preferred play activities and gender of playmates (e.g., Golombok & Rust, 1993; Hines et al., 2002; Hönekopp & Thierfelder, 2009).

Organizational T effects might play a similar role in GD. Transmen might have experienced stronger masculinization than is typical for their natal sex, and the opposite might be true for transwomen. We consider two major lines of research supporting this hypothesis.

### Brain Differences Between People With and Without a History of Gender Dysphoria

The first line of evidence draws on potential differences in the brain structures between transpeople and controls without a history of GD. Reviews (Guillamon, Junque, & Gómez-Gil, 2016; Kreukels & Guillamon, 2016) concluded that transpeople’s brains show some atypical changes away from their natal sex and toward their experienced gender identity. However, independent replications of these findings have not yet been conducted, and therefore some caution is required (e.g., LeBel & Peters, 2011; Open Science Collaboration, 2015).

### Gender Dysphoria in People with Disorders of Sex Development

A second major line of evidence regarding organizational T effects on GD stems from disorders of sex development (DSD). Across a range of DSDs, gender of rearing and presumed masculinization of the brain can be aligned, partly misaligned, or at odds, irrespective of sex chromosomes (Hughes et al., 2006).

Females (46, XX) with congenital adrenal hyperplasia (CAH) produce excessive amounts of androgens as fetuses, which often leads to genital masculinization. In this group, relatively high rates of gender change or GD were observed: 5% among those raised as girls, and 12% among those raised as boys (Callens et al., 2016; Dessens, Slijper, & Drop, 2005; Mattila, Fagerholm, Santtila, Miettinen, & Taskinen, 2012). In individuals raised as girls, the cause might be the comparatively high prenatal T and its effects on brain. In individuals raised as boys, early medical treatment of their condition implies that they did not experience the boy-typical T-surge after birth; consequently, comparatively low perinatal brain masculinization might contribute to the development of a female gender identity.

In 46, XY individuals with partial or complete androgen insensitivity syndrome (AIS/CAIS), testicular androgen production is normal but has little or no effect (Hughes et al., 2012). In CAIS, which typically is only detected in adolescence, female genitalia develop and no perinatal brain masculinization occurs. Thus, the lack of perinatal brain masculinization and gender of rearing are aligned, and no gender change was observed in this group. In contrast, AIS leads to ambiguous genital development, and incomplete perinatal brain masculinization appears plausible. In this group, frequent gender change was observed, regardless of gender of rearing (Callens et al., 2016; Mazur, 2005). This possibly reflects that incomplete perinatal brain masculinization can be at odds with either male or female upbringing and therefore constitutes a risk factor for GD.

In other conditions in which 46, XY individuals undergo normal perinatal brain masculinization but show atypical genital development (penis loss through accidents; micropenis; aphallia; cloacal and classical exstrophy of the bladder), cases of gender change were not observed among individuals raised as boys but occurred frequently among individuals raised as girls (Mazur, 2005; Meyer-Bahlburg, 2005). This supports again the idea that T affects gender identity.

Across DSDs, levels of gender change or GD are consistently high when (inferred) brain androgenization mismatches gender of rearing but consistently low when brain androgenization and gender of rearing match. This supports the idea that organizational T effects impact on GD. However, confounding variables pose potential problems (for contrasting views, see Cohen-Bendahan, van de Beek, & Berenbaum, 2005; Jordan-Young, 2012). Further, GD typically develops in people without DSD, and generalization from the latter to the former might be problematic. For example, gender change in individuals with a DSD does not necessarily reflect GD (Cadet, 2011), and only DSD individuals have a lifelong history in which medical service providers and parents problematize their gender (Meyer-Bahlburg, 2009). Consequently, converging evidence for a role of organizational T effects on gender identity is desirable.

### 2D:4D Digit Ratio

In a classic paper, Manning, Scutt, Wilson, and Lewis-Jones (1998) suggested that low values for digit ratio 2D:4D (i.e., the length of the 2nd digit divided by the length of the 4th digit) reflect high prenatal T activity in humans. A suite of observations suggests that 2D:4D might be a useful measure of prenatal T effects: 2D:4D shows a moderate sex difference across numerous countries studied (Grimbos, Dawood, Burriss, Zucker, & Puts, 2010; Hönekopp & Watson, 2010). This sex difference is established in utero, probably at the end of the first trimester (Galis, Ten Broek, Van Dongen, & Wijnaendts, 2010; Malas, Dogan, Evcil, & Desdicioglu, 2006), but the sex difference appears largely unaffected by puberty (Králík, Ingrová, Kozieł, Hupková, & Klíma, 2017; McIntyre, Ellison, Lieberman, Demerath, & Towne, 2005; Trivers, Manning, & Jacobson, 2006). 2D:4D shows longitudinal stability (Králík et al., 2017; McIntyre, Cohn, & Ellison, 2006; McIntyre, Ellison, Lieberman, Demerath, & Towne, 2005; Wong & Hines, 2016), but see Knickmeyer, Woolson, Hamer, Konneker, and Gilmore (2011) for an exception. Probably the best evidence that 2D:4D reflects prenatal T effects stems from individuals who were exposed to atypical T effects during early development: Females with high prenatal T levels due to CAH have strongly masculinized 2D:4D (Brown, Hines, Fane, & Breedlove, 2002; Hönekopp & Watson, 2010; Kocaman et al., 2017; Rivas et al., 2014); similarly, men affected by Klinefelter’s syndrome, which causes low T levels throughout development, show strongly feminized 2D:4D (Manning, Kilduff, & Trivers, 2013); finally, genetic males affected by CAIS show moderately feminized 2D:4D (Berenbaum, Bryk, Nowak, Quigley, & Moffat, 2009; van Hemmen, Cohen-Kettenis, Steensma, Veltman, & Bakker, 2017).

### Aims

As discussed above, observations from DSDs suggest that the incidence of GD in these conditions is increased when the sex of rearing mismatches early brain masculinization. This observation points to a potential role of prenatal T in the development of GD in individuals unaffected by DSD: strong prenatal T effects in females and weak prenatal T effects in males might increase the risk for GD. To address this hypothesis, we first report the largest sample for expert-measured 2D:4D in transpeople so far. Based on their statistical significance, findings from earlier studies have been interpreted as inconsistent (Manning, 2017). However, comparing studies on their statistical significance is an ill-suited criterion to judge the agreement of results (e.g., Cumming, 2014; Hunter, 1997). Therefore, we then present a meta-analysis of the pertinent evidence here. We hypothesize that transmen show, on average, masculinized (i.e., lower than female-typical) 2D:4D, whereas transwomen show feminized (i.e., higher than male-typical) 2D:4D.

## The Mashhad Study

### Method

#### Participants

Between January 2015 and December 2016, 203 individuals with GD were consecutively referred to the Transgender Studies Centre, at Mashhad University of Medical Sciences, in Mashhad, Iran. The diagnostic process of GD was largely based on the Standards of Care, version 7 of the World Professional Association of Transgender Health (WPATH) (Coleman et al., 2012). All individuals had been interviewed by at least two experienced psychiatrists according to DSM-5 criteria, and the GD diagnosis was confirmed for all participants in this group. The interviews also assessed participants’ sexual orientation, their history of GD, and their childhood play behavior. Those who reported identifying as a member of the other sex and/or sex incongruent behaviors before puberty were classified as “early onset”; the other participants were classified as “late onset.” An endocrinologist examined all participants with GD for DSDs, which was ruled out in all cases. Also, none of these participants had been treated with sex hormones.

To limit the effect of potential confounders, potential transsexual participants were screened with regard to a number of exclusion criteria (psychiatric comorbidity, medical disorders including hormonal and chromosomal abnormalities, and a history of finger fracture or dislocation). Three potential participants were excluded for a possible diagnosis of schizophrenia; six with bipolar disorder; and one with thalassemia. A total of 117 medical students and staff at Ibne-sina Hospital and Imam-reza Hospital volunteered as cisgender participants. Absence of the same exclusion criteria that were applied in the transsexual group was confirmed. 2D:4D ratios could not be obtained for one transwoman and nine controls because of obscured or ambiguous landmarks for finger lengths. We performed analyses on the remaining 104 transmen (natal females; *M* age 25.3 years, SD = 6.2; 103 gynephilic; 92 early onset), 89 transwomen (natal males; *M* = 26.0 years, SD = 6.6; all androphilic; 81 early onset), 53 control females (*M* = 24.9 years, SD = 4.5), and 56 control males (*M* = 28.9 years, SD = 9.5).

The study was approved by the ethics committee of Mashhad University of Medical Sciences. We explained the purpose of the study to the participants, and an informed written consent was obtained.

#### Measures

The palmar surface of the right and left hand of all participants was photocopied. Participants stretched their fingers and applied minimal pressure to the glass plate. All 2D and 4D lengths were then measured with digital vernier calipers from the digit tip to the middle of its most proximal crease. To check the reliability of the resulting 2D:4D, the photocopies of 37 hands were digitized and their 2D and 4D lengths were independently re-measured. The resulting two series of 2D:4D correlated satisfactorily, *r* = 86. All measurements were performed blind to participants’ gender identity.

#### Open Materials

The publication of data is widely regarded as an important step for safeguarding the validity of published research in psychology (e.g., Cumming, 2014; Munafò et al., 2017; Shrout & Rodgers, 2018). Therefore, all data are available at https://osf.io/jtyf4/.

### Results and Discussion

**Fig. 1:**
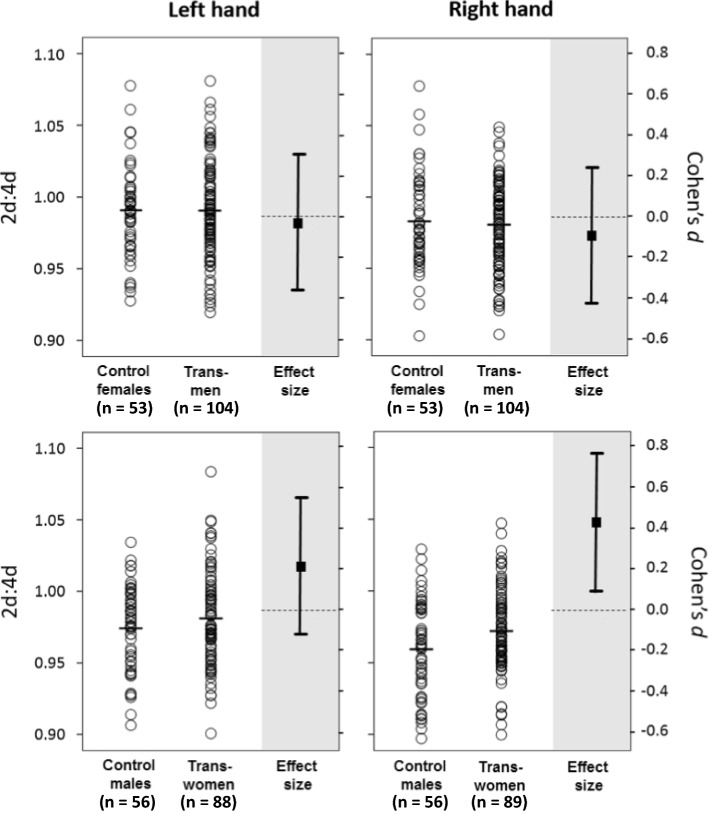
Digit ratio 2D:4D in transgender versus cisgender participants. *Note*: Individual 2D:4D scores (open circles against white background) are read against the left-hand ordinates; horizontal markers indicate group means. Standardized mean differences (filled square against gray background) are read against the right-hand ordinates; positive values indicate that mean 2D:4D was higher in transpeople than in controls, and error bars indicate 95% CIs

Table 1 provides descriptive statistics for 2D:4D. A preliminary 2 (Sex: Female vs. Male) × 2 (Hand: Left vs. Right) mixed ANOVA on 2D:4D that included only control participants revealed the expected normative effect of gender on 2D:4D, *F*(1, 107) = 12.64, *p* = .001. In order to compare 2D:4D between transsexuals and controls of the same natal sex, we ran a 2 (Group: Transsexual vs. Cisgender) × 2 (Hand: Right vs. Left) mixed ANOVA separately on natal males and natal females. Group proved statistically significant for natal males, *F*(1, 142) = 4.33, *p* = .001, indicating lower (less masculine) 2D:4D in transwomen compared to control men. Group did not prove statistically significant for natal females, *F*(1, 155) = 0.07, *p* = .789. Figure 1 provides separate comparisons for each hand with standardized effect sizes.

**Table 1:** Means (and SD) for 2D:4D in the left and right hand for transmen, transwomen, control women, and control men

	Transmen	Control women	Transwomen	Control men
Left 2D:4D	0.991 (0.034)	0.991 (0.032)	0.981 (0.033)	0.974 (0.029)
	*n* = 104	*n* = 53	*n* = 88	*n* = 56
Right 2D:4D	0.981 (0.030)	0.983 (0.033)	0.972 (0.029)	0.959 (0.033)
	*n* = 104	*n* = 53	*n* = 89	*n* = 56

Sexual orientation and age of onset have been suggested to differentiate subtypes of transpeople, and these variables might indicate potential differences in GD etiology (Lawrence, 2010, 2017). Sexual orientation has also been linked to 2D:4D (e.g., Breedlove, 2017, 2019; Grimbos et al., 2010; McFadden et al., 2005; Williams et al., 2000). Both of our transpeople samples lacked variation in sexual orientation, thus precluding analyses of this factor. However, we could investigate the effect of GD onset, for which Table 2 shows descriptive statistics. We ran separate 2 (GD onset: Early vs. Late) × 2 (Hand: Right vs. Left) mixed ANOVAs on transwomen and transmen. In transwomen, neither GD onset nor its interaction with hand proved statistically significant (*p* ≥ .40). But in transmen, GD onset proved statistically significant, *F*(1, 102) = 8.55, *p* = .004); transmen with early GD onset (*N* = 92) had lower (more masculine) 2D:4D than those with late onset (*N* = 12). This difference proved large in the right hand, *d* = 1.04, 95%CI [0.42, 1.66] and medium-sized in the left hand, *d* = 0.62 [0.02, 1.23]. Given the small number of transmen with late onset and the exploratory nature of this analysis, some caution seems appropriate. Nonetheless, these results lend some support to the idea that different GD subtypes can be differentiated (Lawrence, 2010, 2017).

**Table 2:** Means (and SD) for 2D:4D in transsexuals’ left and right hand as a function of early or late onset of gender dysphoria

	Transwomen		Transmen	
	Early onset	Late onset	Early onset	Late onset
Left 2D:4D	0.982 (0.034)	0.975 (0.022)	0.988 (0.033)	1.009 (0.031)
	*n* = 80	*n* = 8	*n* = 92	*n* = 12
Right 2D:4D	0.973 (0.029)	0.963 (0.026)	0.977 (0.028)	1.007 (0.027)
	*n* = 81	*n* = 8	*n* = 92	*n* = 12

## Meta-Analysis of 2D:4D in Transsexuals

### Method

#### Study Retrieval and Inclusion Criteria

Childhood GD (unlike transsexualism) is often transient (e.g., Drummond, Bradley, Peterson-Badali, & Zucker, 2008). We therefore focused exclusively on adult samples. To locate relevant previous studies, we searched the topic field in Web of Science for *2D:4D* or *digit ratio* in conjunction with either transsex* or *transgender* or *gender dysphoria* or *gender identity disorder* or *sex reassignment*. This led to 19 hits. Closer scrutiny identified eight relevant studies that compared 2D:4D between transpeople and controls with the same natal sex. Examination of their reference sections did not uncover any additional studies.

In order to be suitable for our meta-analysis, information for the calculation of a standardized mean difference (*d*) needed to be available for at least one natal sex (e.g., descriptive statistics, suitable test statistics or *p* values, figures with error bars). For five reports, relevant information was not (fully) available. We emailed corresponding authors for additional information and repeated this once when we received no response to our initial message. In this way, we could include six previous studies in our meta-analysis (Hisasue, Sasaki, Tsukamoto, & Horie, 2012; Kraemer et al., 2009; Leinung & Wu, 2017; Mas et al., 2009; Schneider, Pickel, & Stalla, 2006; Wallien, Zucker, Steensma, & Cohen-Kettenis, 2008). Two studies had to be excluded due to incomplete or contradictory information.

#### Analyses

We used *d* as our effect size measure. As in our Mashhad study, positive effect sizes indicated higher average 2D:4D in transpeople than in controls of the same natal sex (expected in transwomen), whereas negative effect sizes indicate lower average 2D:4D in transpeople (expected in transmen).

For each of the four combinations of natal sex by hand, we ran a separate random-effects meta-analysis. Two central outcome measures are of particular interest (Schmidt, Oh, & Hayes, 2009): The first, *d*, is simply the pooled estimate for the unknown population effect size *δ* (with values of 0.2/0.5/0.8 typically considered small/medium/large in psychology). The second outcome measure, *T*, reflects heterogeneity in the results and is important for the appropriate interpretation of *d*. Even when a study is closely replicated with different random samples from the same population, the resulting study effect sizes will vary, thus reflecting the randomness inherent in sampling. Heterogeneity emerges when the observed variability in study effect sizes exceeds the level expected from sampling alone. Typically, heterogeneity indicates that the question “What is the true population effect size?” cannot be answered with a single figure (e.g., “we estimate *δ* = 0.5”). The effectiveness of a drug, for example, might depend on the selected dose, on patients’ sex, age, diet, and genetic make-up, on their concurrent medical conditions, etc. Consequently, a set of efficacy studies using different regimes and patient groups should demonstrate heterogeneity. Therefore, the answer to “how effective is the drug?” should be “it depends…” The true effectiveness of the drug cannot be sensibly described with a single effect size, but only as a distribution of effect sizes. *d* estimates the mean of this distribution and *T* (our measure of heterogeneity) estimates its standard deviation. For example, *d* = 0.5 and *T* = 0.1 would indicate that the drug’s true effectiveness varies under most circumstances within a moderate band (from *δ* = 0.3 to *δ* = 0.7 if we use *M* ± 2SD); but for *d* = 0.5 and *T* = 0.4 we would infer that effectiveness varies so widely that it includes systematic harm (from *δ* = − 0.3 to *δ* = 1.3). Similarly, in our meta-analyses *T* > 0 suggests that the respective primary studies tapped into populations that differ in true effect size. For both *d* and *T*, significance tests can indicate if they deviate more strongly from zero than expected by chance alone. We performed all analyses with the R package *metafor* (Viechtbauer, 2010). Again, all materials and data are available at https://osf.io/jtyf4/.

### Results and Discussion

For left hands, our meta-analyses were based on 252 transmen with 440 female controls and on 353 transwomen with 488 male controls, with *k* = 5 samples in each case. For right hands, our meta-analyses were based on 301 transmen with 456 female controls and on 420 transwomen with 505 male controls, with *k* = 6 samples in each case.

**Fig. 2:**
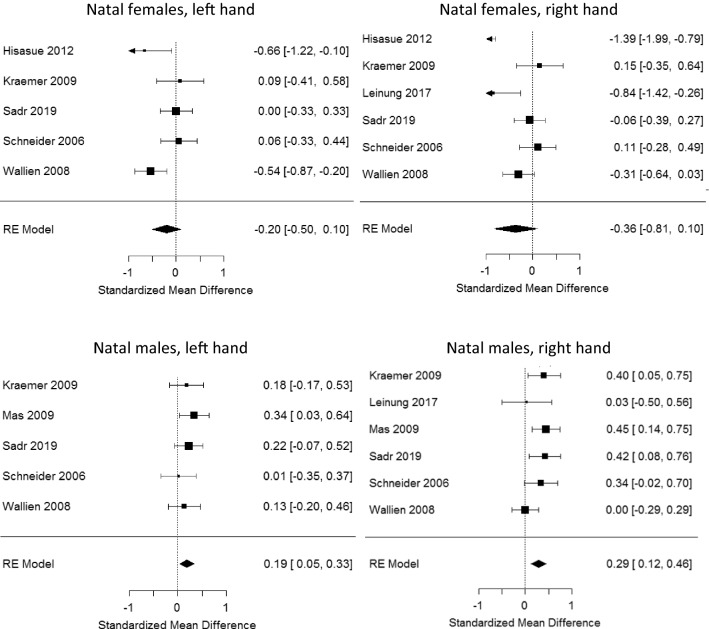
Four meta-analyses comparing 2D:4D in transgender and cisgender participants of the same natal sex. *Note*: The top part in each panel shows individual study effect sizes and their 95% CIs; the bottom part shows, for each random-effects meta-analysis, the overall effect size with its 95% CI. Negative effect sizes (masculinized 2D:4D in transmen) were hypothesized for the top panels; positive effect sizes (feminized 2D:4D in transwomen) were hypothesized for the bottom panels

Primary study effect sizes and meta-analytic results are shown in Fig. 2. As expected, the two meta-analyses for transmen showed negative effects (masculinized 2D:4D) (see top panels in Fig. 2). However, the effect sizes (*d* = − .20 for left hands and *d* = − .36 for right hands) were not statistically significant (left hand: *p* = .195; right hand: *p* = .123).

Strong heterogeneity was observed for both the left hand (*T* = 0.27, *p* = .028) and the right hand (*T* = 0.52, *p* = .0002). Manning (2017) suggested that clearer results emerge when digit lengths are not measured from photocopies or scans but directly on the fingers. We could test this idea for right hands. Type of measurement did not emerge as a statistically significant moderator, *Q*(1) = 0.72, *p* = .398. However, given that only one study (Leinung & Wu, 2017) used direct measurements, statistical power was presumably low. When both meta-analyses were repeated without the largest effect (Hisasue et al., 2012), heterogeneity remained large and statistically significant for both hands. We are not aware of any factors that would explain the large observed heterogeneity.

As expected, the two meta-analyses for transwomen showed positive effects (feminized 2D:4D) (see bottom panels in Fig. 2). Both effects were small, but statistically significant (left hand: *d* = 0.19, *p* = .010; right hand: *d* = 0.29, *p* = .0009). For left hands, no heterogeneity was observed (*T* = 0). For right hands, some heterogeneity was observed (*T* = 0.11). However, this was not statistically significant, *Q*(5) = 6.8, *p* = .237; thus, chance on its own would be fairly likely to generate the moderate level of heterogeneity observed. For right hands, we could again test Manning’s (2017) idea that type of finger measurement moderates results. Again, no such evidence emerged, *Q*(1) = 0.81, *p* = .370. But again, statistical power must be presumed to be low because only Leinung and Wu (2017) used direct measurements.

A preference for the publication of statistically significant studies can cause a systematic bias in the published literature (e.g., Sterling, 1959), which is a natural concern for meta-analysis (and any other type of literature review). Tests for this kind of publication bias have low statistical power when the number of primary studies is small (Ioannidis, 2008), as was the case here. We therefore did not perform any formal tests. However, as Fig. 2 shows, the results of most primary studies were not statistically significant on their own. (This is indicated by 95% CIs containing zero.) If a powerful publication bias had shaped the literature we reviewed here, this pattern of results would not be expected (e.g., Francis, Tanzman, & Matthews, 2014). Consequently, we believe that publication bias is not a concern in the body of literature reviewed here.

Although transsexualism and sexual orientation are distinct, they are not independent: Sexual attraction to women is much more common among transmen than among non-transsexual natal females; similarly, sexual attraction to men is much more common among transwomen than among non-transsexual natal males (e.g., Wallien et al., 2008). It is therefore interesting to note parallels and differences regarding their relationship with 2D:4D. Whereas our meta-analysis established a clear link between 2D:4D and transsexualism for natal males only, the opposite holds for sexual orientation. A meta-analysis found a link with 2D:4D for females only (Grimbos et al., 2010). As expected, lesbians showed lower (masculinized) 2D:4D than heterosexual women, with a small-to-medium effect size similar to the one we observed for natal males.

One limitation of our meta-analyses is that, due to insufficient information in the primary studies, we could not include data on 495 transwomen and 160 transmen. Given the observed homogeneity for results in transwomen, it seems plausible that these additional data would not change the overall picture in this group. More importantly, the discussions of the relevant reports suggest that the observed effects were in line with expectations (Veale, Clarke, & Lomax, 2010; Vujović et al., 2014). We therefore think it is fair to conclude that inclusion of these effects would not have substantially changed our findings.

Regrettably, a lack of detailed information in the primary studies precluded more fine-grained analyses of sexual orientation and age of GD onset, which might characterize distinct subtypes of transwomen and transmen (Lawrence, 2010, 2017). We hope that future studies will address these variables in greater detail.

## General Discussion

As we discussed in the introduction, the pattern of GD rates across DSDs suggests a role for prenatal T in GD development. More specifically, a mismatch between perinatal brain masculinization and sex of rearing appears to increase GD risk. Similarly, a relatively high degree of masculinization of the brain in females and a relatively low degree of masculinization of the brain in males might increase GD risk in individuals unaffected by DSDs. Studies in people with DSDs cannot address the latter question, and atypical perinatal T effects in DSDs are confounded with a lifelong history in which medical service providers and parents problematize gender. For these reasons, convergent evidence beyond observations in DSDs is desirable to corroborate any role of perinatal T in GD. Digit ratio 2D:4D, an index of prenatal T effects, strikes us as a suitable tool.

Here, we presented the largest sample for expert-measured 2D:4D in transpeople and a meta-analysis of pertinent 2D:4D studies. As a mix of statistically significant and non-significant findings in individual studies might easily mask a clear pattern of results (e.g., Hunter, 1997), we focus our discussion on the meta-analysis. For transwomen, a clear pattern emerged. In line with our hypothesis, transwomen showed feminized (higher) 2D:4D. The effect was small, but consistent across studies and across both hands. For transmen, the evidence was more tentative: In both hands, transmen showed masculinized (lower) 2D:4D, and the average effects were slightly stronger than for transwomen. However, large heterogeneity between studies was observed and rendered the observed mean effects not statistically significant (Schmidt et al., 2009). Additional studies might increase statistical power and thus turn the overall effect statistically significant; however, it seems unlikely that heterogeneity would disappear. The studies analyzed here stem from markedly different cultures, among them Iran, Japan, and Switzerland. The observed heterogeneity in effect sizes might reflect that the role of prenatal androgenization for the development of GD in natal females differs across cultures.

Our results suggest that weak prenatal T effects in natal males contribute to GD risk. The meta-analyses tentatively suggest that strong prenatal T effects in natal females increase GD risk under circumstances yet to be identified.

### Convergent Results from CAH and Digit Ratio Beyond Gender Dysphoria Underpin the Validity of 2D:4D

In the introduction, we discussed various strands of evidence that 2D:4D tracks prenatal T effects. Perhaps the strongest comes from observations in DSDs and other conditions in which atypical prenatal T effects are reliably accompanied by analogous changes in 2D:4D (Berenbaum et al., 2009; Brown, Finn, Cooke, & Breedlove, 2002; Kocaman et al., 2017; Manning et al., 2013; Rivas et al., 2014; van Hemmen et al., 2017). Some researchers, though, called the validity of 2D:4D into question and argued that it is unsuitable for studying perinatal T effects (Berenbaum & Beltz, 2016; Hines et al., 2015). We address this criticism in two ways. In this section, we review to what extent 2D:4D studies do or do not converge with CAH studies (which critics of 2D:4D regard as particularly useful for studying prenatal T effects) beyond the domain of GD. After that, we look in greater detail at the arguments levelled against 2D:4D and use them to discuss the strengths and limitations of our current findings.

Observations from CAH-affected females offer particularly strong evidence that gendered play behavior is affected by prenatal T (Berenbaum & Beltz, 2016; Hines et al., 2015). A number of studies had parents describe their child’s play behavior with the Pre-School Activities Inventory (Golombok & Rust, 1993), which asks for the popularity of various play activities, in order to investigate the relationships with 2D:4D (Hönekopp & Thierfelder, 2009; Körner, Pause, & Heil, 2017; Mitsui et al., 2016; Wong & Hines, 2016). Analyses were performed separately for girls and boys. Almost all correlations were in the expected direction (indicating that more feminine 2D:4D tended to go with more female-typical play), typically showing small-to-medium effects. Six out of 16 effects proved statistically significant. 2D:4D studies therefore offer considerable support for an effect of prenatal T on gendered play behavior in children. Thus, evidence from CAH studies and 2D:4D studies converges, although CAH studies found much larger effect sizes (Berenbaum & Beltz, 2016).

Another area of interest is autism spectrum disorder, for which prenatal T is believed to be a risk factor (Baron-Cohen, 2002). In line with this idea, a meta-analysis found clear evidence for lower (masculinized) 2D:4D in individuals affected by autism spectrum disorder (Hönekopp, 2012), with a medium effect size and no clear sign for heterogeneity in results (see also Al-Zaid, Alhader, & Al-Ayadhi, 2015, for a later study with similar results). Investigations whether autism risk is increased in CAH-affected individuals are hardly feasible because both conditions are rare. However, three studies investigated if autism-typical symptoms tend to be elevated in CAH-affected individuals (Knickmeyer et al., 2006; Kocaman et al., 2017; Kung et al., 2016). Across three female and two male samples, four effects pointed in this direction (*d* = 0.30 to *d* = 0.54), with two of them being statistically significant; one male sample found a small, statistically non-significant effect in the opposite direction (*d* = − 0.25, Kung et al., 2016). Given their different outcome measures (diagnosis of autism spectrum disorder versus degree of autism-typical symptoms), 2D:4D studies and CAH studies are difficult to compare directly; nonetheless, both garnered evidence that elevated prenatal T increases autism risk.

Another area of interest is sexual orientation. A review found that all eight pertinent studies reported higher bisexual/homosexual orientation in CAH-affected women than in unaffected controls (Meyer-Bahlburg, Dolezal, Baker, & New, 2008). This suggests that strong prenatal T effects shift female sexual behavior and fantasies in a male-typical direction. In line with this, a meta-analysis of studies comparing 2D:4D in homosexual and heterosexual individuals found lower (masculinized) 2D:4D in lesbians (Grimbos et al., 2010); no effect was observed for gay versus heterosexual men. Whereas the effect size for masculinized 2D:4D in lesbians was small, rates of bisexual/homosexual orientation in women with CAH were often substantially increased.

A final area of interest is aggression. Findings on aggression in CAH-affected females appear somewhat inconsistent. In comparison with controls, females with CAH have been reported to show statistically significantly lower levels of aggression (Helleday, Edman, Ritzén, & Siwers, 1993), about the same levels of aggression (e.g., Berenbaum & Resnick, 1997; Money & Schwartz, 1976), and statistically significantly higher levels of aggression (Berenbaum & Resnick, 1997; Pasterski et al., 2007). Overall, however, results appear to lean toward greater aggression in CAH-affected females. Two pertinent meta-analyses on 2D:4D studies found no analogous link with aggression in females (and at best tentative evidence for a relationship in males, cf. Hönekopp & Watson, 2011; Turanovic, Pratt, & Piquero, 2017). Therefore, results from CAH studies and from 2D:4D do not converge when it comes to aggression. Two points are of note though. First, the evidence from CAH studies appears less conclusive than is the case for GD, play behavior, autism-typical symptoms, and sexual orientation. Second, CAH studies into aggression typically rely on healthy female controls although chronically ill controls might be more appropriate, because the burdens of illness might increase aggression; we are aware of only one study with such a control group, and it presented no evidence for greater aggression in CAH-affected females (Slijper, 1984).

In sum, there is broad convergence in the results of CAH studies and 2D:4D studies: Both provide evidence for prenatal T effects on GD, gendered play behavior, autism, and sexual orientation; however, aggression is a potential exception. It should be noted though that effect sizes tend to be considerably larger in CAH studies than in 2D:4D studies whenever the use of similar outcome measures makes such comparisons meaningful. A broader review that goes beyond CAH studies to investigate convergence between 2D:4D studies and studies using other means to investigate prenatal T effects would be desirable, but is beyond the scope of our paper.

### Strengths and Limitations of 2D:4D

Some researchers have argued against the usefulness of 2D:4D for studying prenatal T effects in humans, primarily because 2D:4D appears to be a noisy measure (e.g., Berenbaum & Beltz, 2016; Hines et al., 2015). For example, a large difference in prenatal T effects between typically developing males and 46, XY individuals with CAIS translates into 2D:4D distributions that show strong overlap between both groups (Berenbaum et al., 2009). The general pattern discussed earlier—2D:4D studies tend to find smaller effect sizes than comparable CAH studies—points in the same direction. However, the value of a noisy measure depends on the relative merits and limitations of available alternatives. For example, the easy availability of 2D:4D means that it can be used to study small populations of interest that are difficult to address via CAH, amniocentesis, or other means that address prenatal T directly (e.g., Coates, Gurnell, & Rustichini, 2009; Manning, Baron-Cohen, Wheelwright, & Sanders, 2001; Voracek, Reimer, Ertl, & Dressler, 2006). Possibly owing to ease of measurement, 2D:4D studies have also looked at a much broader range of outcome measures than has been achieved with alternative means for studying prenatal T effects. For example, negative correlations between 2D:4D and physical performance are well established (e.g., Hönekopp, Manning, & Müller, 2006; Manning, 2002). These relationships are strong when performance hinges upon endurance but absent or weak when performance hinges on strength (Hönekopp & Schuster, 2010), and a detailed understanding of these relationships at a physiological level might be within reach (Holzapfel, Chomentowski, Summers, & Sabin, 2016). Naturally, convergent evidence for prenatal T effects on endurance from future studies relying on different methods would be desirable. In general, our meta-analysis on transpeople and our review of other domains demonstrates that 2D:4D and other avenues for studying prenatal T can sensibly complement each other.

Although sex of rearing seems to powerfully shape gender identity, rates of GD are strongly elevated in a number of DSDs (Callens et al., 2016; Dessens et al., 2005; Mazur, 2005). Among other predictors for a positive outcome, degree of prenatal T effects can play a role in gender assignment after birth (Hughes et al., 2006). Nonetheless, it seems unlikely that 2D:4D can meaningfully contribute to treatment planning: Given that it is probably a noisy measure of individual prenatal T effects (Breedlove, 2010), 2D:4D can only be a weak predictor of outcome quality, which encompasses physical health, fertility, well-being, and other facets. And adding a weak predictor to one or more existing stronger predictors usually does not increase prediction accuracy (Cohen, 1990; Dawes, 1979; Gigerenzer & Goldstein, 1999).

However, 2D:4D advances our understanding of GD. The higher 2D:4D observed in our meta-analyses for transwomen suggests that low prenatal T levels increases GD risk in natal males. It also found some evidence that high prenatal T levels increase GD risk in natal females. Overall, prenatal T effects appear to play a small role in GD development. Future studies might show if early postnatal T has similar effects, and what other factors increase GD risk.

## References

[CR1] Al-Zaid FS, Alhader AA, Al-Ayadhi LY (2015). The second to fourth digit ratio (2D:4D) in Saudi boys with autism: A potential screening tool. Early Human Development.

[CR2] American Psychiatric Association (2013). Diagnostic and statistical manual of mental disorders.

[CR3] Baron-Cohen S (2002). The extreme male brain theory of autism. Trends in Cognitive Sciences.

[CR4] Berenbaum SA, Beltz AM (2016). How early hormones shape gender development. Current Opinion in Behavioral Sciences.

[CR5] Berenbaum SA, Bryk KK, Nowak N, Quigley CA, Moffat S (2009). Fingers as a marker of prenatal androgen exposure. Endocrinology.

[CR6] Berenbaum SA, Resnick SM (1997). Early androgen effects on aggression in children and adults with congenital adrenal hyperplasia. Psychoneuroendocrinology.

[CR7] Breedlove SM (2010). Minireview: Organizational hypothesis: Instances of the fingerpost. Endocrinology.

[CR8] Breedlove SM (2017). Prenatal influences on human sexual orientation: Expectations versus data. Archives of Sexual Behavior.

[CR9] Breedlove SM (2019). Replicable data for digit ratio differences. Science.

[CR10] Brown WM, Finn CJ, Cooke BM, Breedlove SM (2002). Differences in finger length ratios between self-identified “butch” and “femme” lesbians. Archives of Sexual Behavior.

[CR11] Brown WM, Hines M, Fane BA, Breedlove SM (2002). Masculinized finger length patterns in human males and females with congenital adrenal hyperplasia. Hormones and Behavior.

[CR12] Cadet P (2011). Androgen insensitivity syndrome with male sex-of-living [Letter to the Editor]. Archives of Sexual Behavior.

[CR13] Callens N, Van Kuyk M, van Kuppenveld JH, Drop SL, Cohen-Kettenis PT, Dessens AB, on behalf of the Dutch Study Group on DSD (2016). Recalled and current gender role behavior, gender identity and sexual orientation in adults with disorders/differences of sex development. Hormones and Behavior.

[CR14] Coates JM, Gurnell M, Rustichini A (2009). Second-to-fourth digit ratio predicts success among high-frequency financial traders. Proceedings of the National Academy of Sciences.

[CR15] Cohen J (1990). Things I have learned (so far). American Psychologist.

[CR16] Cohen-Bendahan CC, van de Beek C, Berenbaum SA (2005). Prenatal sex hormone effects on child and adult sex-typed behavior: Methods and findings. Neuroscience and Biobehavioral Reviews.

[CR17] Coleman E, Bockting W, Botzer M, Cohen-Kettenis P, DeCuypere G, Feldman J, Fraser L, Green J, Knudson G, Meyer WJ, Monstrey S, Adler RK, Brown GR, Devor AH, Ehrbar R, Ettner R, Eyler E, Garofalo R, Karasic DH, Lev AI, Mayer G, Meyer-Bahlburg H, Hall BP, Pfaefflin F, Rachlin K, Robinson B, Schechter LS, Tangpricha V, van Trotsenburg M, Vitale A, Winter S, Whittle S, Wylie KR, Zucker K (2012). Standards of care for the health of transsexual, transgender, and gender-nonconforming people, version 7. International Journal of Transgenderism.

[CR18] Cumming G (2014). The new statistics: Why and how. Psychological Science.

[CR19] Dawes RM (1979). The robust beauty of improper linear models in decision making. American Psychologist.

[CR20] Dessens AB, Slijper FM, Drop SL (2005). Gender dysphoria and gender change in chromosomal females with congenital adrenal hyperplasia. Archives of Sexual Behavior.

[CR21] Drummond KD, Bradley SJ, Peterson-Badali M, Zucker KJ (2008). A follow-up study of girls with gender identity disorder. Developmental Psychology.

[CR22] Francis G, Tanzman J, Matthews WJ (2014). Excess success for psychology articles in the journal science. PLoS ONE.

[CR23] Galis F, Ten Broek CM, Van Dongen S, Wijnaendts LC (2010). Sexual dimorphism in the prenatal digit ratio (2D:4D). Archives of Sexual Behavior.

[CR24] Gigerenzer G, Goldstein DG, Gigerenzer G, Todd PM, ABC Research Group (1999). Betting on one good reason: The take the best heuristic. Simple heuristics that make us smart.

[CR25] Golombok S, Rust J (1993). The Pre-School Activities Inventory: A standardized assessment of gender role in children. Psychological Assessment.

[CR26] Grimbos T, Dawood K, Burriss RP, Zucker KJ, Puts DA (2010). Sexual orientation and the second to fourth finger length ratio: A meta-analysis in men and women. Behavioral Neuroscience.

[CR27] Guillamon A, Junque C, Gómez-Gil E (2016). A review of the status of brain structure research in transsexualism. Archives of Sexual Behavior.

[CR28] Helleday J, Edman G, Ritzén EM, Siwers B (1993). Personality characteristics and platelet MAO activity in women with congenital adrenal hyperplasia (CAH). Psychoneuroendocrinology.

[CR29] Hines M (2010). Sex-related variation in human behavior and the brain. Trends in Cognitive Sciences.

[CR30] Hines, M., Constantinescu, M., & Spencer, D. (2015). Early androgen exposure and human gender development. *Biology of Sex Differences,**6*. 10.1186/s13293-015-0022-1. 10.1186/s13293-015-0022-1 PMC4350266 25745554

[CR31] Hines M, Johnston KJ, Golombok S, Rust J, Stevens M, Golding J, Team AS (2002). Prenatal stress and gender role behavior in girls and boys: A longitudinal, population study. Hormones and Behavior.

[CR32] Hisasue SI, Sasaki S, Tsukamoto T, Horie S (2012). The relationship between second-to-fourth digit ratio and female gender identity. Journal of Sexual Medicine.

[CR33] Holzapfel, S., Chomentowski III, P., Summers, L., & Sabin, M. (2016). 2D:4D and aerobic fitness in young adults: The relationship between digit ratio (2D:4D), VO2max, ventilatory threshold, and running performance. *International Journal of Sports Sciences & Fitness, 6*(1).

[CR34] Hönekopp J (2012). Digit ratio 2D:4D in relation to autism spectrum disorders, empathizing, and systemizing: A quantitative review. Autism Research.

[CR35] Hönekopp J, Manning JT, Müller C (2006). Digit ratio (2D:4D) and physical fitness in males and females: Evidence for effects of prenatal androgens on sexually selected traits. Hormones and Behavior.

[CR36] Hönekopp J, Schuster M (2010). A meta-analysis on 2D:4D and athletic prowess: Substantial relationships but neither hand out-predicts the other. Personality and Individual Differences.

[CR37] Hönekopp J, Thierfelder C (2009). Relationships between digit ratio (2D:4D) and sex-typed play behavior in pre-school children. Personality and Individual Differences.

[CR38] Hönekopp J, Watson S (2010). Meta-analysis of digit ratio 2D:4D shows greater sex difference in the right hand. American Journal of Human Biology.

[CR39] Hönekopp J, Watson S (2011). Meta-analysis of the relationship between digit-ratio 2D:4D and aggression. Personality and Individual Differences.

[CR40] Hughes IA, Davies JD, Bunch TI, Pasterski V, Mastroyannopoulou K, MacDougall J (2012). Androgen insensitivity syndrome. The Lancet.

[CR41] Hughes IA, Houk C, Ahmed SF, Lee PA, Lawson Wilkins Pediatric Endocrine Society (LWPES)/European Society for Paediatric Endocrinology (ESPE) Consensus (2006). Consensus statement on management of intersex disorders. Journal of Pediatric Urology.

[CR42] Hunter JE (1997). Needed: A ban on the significance test. Psychological Science.

[CR43] Ioannidis J (2008). Interpretation of tests of heterogeneity and bias in meta-analysis. Journal of Evaluation in Clinical Practice.

[CR44] Jordan-Young RM (2012). Hormones, context, and “brain gender”: A review of evidence from congenital adrenal hyperplasia. Social Science and Medicine.

[CR45] Khorashad BS, Aghili Z, Kreukels BPC, Hiradfar M, Roshan GM, Afkhamizadeh M, Abbaszadegan MR, Ghaemi N, Khazai B, Cohen-Kettenis PT (2016). Psychosexual outcome among Iranian individuals with 5alpha-reductase deficiency type 2 and its relationship with parental sexism. Journal of Sexual Medicine.

[CR46] Khorashad BS, Roshan GM, Reid AG, Aghili Z, Hiradfar M, Afkhamizadeh M, Talaei A, Aarabi A, Ghaemi N, Taghehchian N, Saberi H, Farahi N, Abbaszadegan MR (2017). Sexual orientation and medical history among Iranian people with complete androgen insensitivity syndrome and congenital adrenal hyperplasia. Journal of Psychosomatic Research.

[CR47] Knickmeyer R, Baron-Cohen S, Fane BA, Wheelwright S, Mathews GA, Conway GS, Brook CG, Hines M (2006). Androgens and autistic traits: A study of individuals with congenital adrenal hyperplasia. Hormones and Behavior.

[CR48] Knickmeyer RC, Woolson S, Hamer RM, Konneker T, Gilmore JH (2011). 2D:4D ratios in the first 2 years of life: Stability and relation to testosterone exposure and sensitivity. Hormones and Behavior.

[CR49] Kocaman GM, Ozmerdivenli R, Yektas C, Bolu S, Haskilic YE, Erdogan A (2017). Autistic feature and 2D:4D finger ratio relations children and adolescents with congenital adrenal hyperplasia. Anadolu Psikiyatri Dergesi-Anatolian Journal of Psychiatry.

[CR50] Körner, L. M., Pause, B. M., & Heil, M. (2017). *Girls play with dolls, boys play with cars*—*Caused by their prenatal testosterone levels?* Paper presented at the TeaP Conference of Experimental Psychologists, Dresden.

[CR51] Kraemer B, Noll T, Delsignore A, Milos G, Schnyder U, Hepp U (2009). Finger length ratio (2D:4D) in adults with gender identity disorder. Archives of Sexual Behavior.

[CR52] Králík M, Ingrová P, Kozieł S, Hupková A, Klíma O (2017). Overall trends vs. individual trajectories in the second-to-fourth digit (2D:4D) and metacarpal (2M:4M) ratios during puberty and adolescence. American Journal of Physical Anthropology.

[CR53] Kreukels BP, Guillamon A (2016). Neuroimaging studies in people with gender incongruence. International Review of Psychiatry.

[CR54] Kung KT, Spencer D, Pasterski V, Neufeld S, Glover V, O’Connor TG, O’Connor TG, Hindmarsh PC, Hughes HA, Acerini CL, Hines M (2016). No relationship between prenatal androgen exposure and autistic traits: Convergent evidence from studies of children with congenital adrenal hyperplasia and of amniotic testosterone concentrations in typically developing children. Journal of Child Psychology and Psychiatry.

[CR55] Lamminmäki A, Hines M, Kuiri-Hänninen T, Kilpeläinen L, Dunkel L, Sankilampi U (2012). Testosterone measured in infancy predicts subsequent sex-typed behavior in boys and in girls. Hormones and Behavior.

[CR56] Lawrence AA (2010). Sexual orientation versus age of onset as bases for typologies (subtypes) for gender identity disorder in adolescents and adults. Archives of Sexual Behavior.

[CR57] Lawrence AA (2017). Autogynephilia and the typology of male-to-female transsexualism. European Psychologist.

[CR58] LeBel EP, Peters KR (2011). Fearing the future of empirical psychology: Bem’s (2011) evidence of psi as a case study of deficiencies in modal research practice. Review of General Psychology.

[CR59] Leinung M, Wu C (2017). The biological basis of transgender identity: 2D:4D finger length ratios implicate a role for prenatal androgen activity. Endocrine Practice.

[CR60] Malas MA, Dogan S, Evcil EH, Desdicioglu K (2006). Fetal development of the hand, digits and digit ratio (2D:4D). Early Human Development.

[CR61] Manning J (2002). The ratio of 2nd to 4th digit length and performance in skiing. Journal of Sports Medicine and Physical Fitness.

[CR62] Manning JT (2017). Prenatal sex steroids and transgender identity: Is there a link with digit ratio?. Endocrine Practice.

[CR63] Manning JT, Baron-Cohen S, Wheelwright S, Sanders G (2001). The 2nd to 4th digit ratio and autism. Developmental Medicine and Child Neurology.

[CR64] Manning JT, Kilduff LP, Trivers R (2013). Digit ratio (2D:4D) in Klinefelter’s syndrome. Andrology.

[CR65] Manning JT, Scutt D, Wilson J, Lewis-Jones DI (1998). The ratio of 2nd to 4th digit length: A predictor of sperm numbers and concentrations of testosterone, luteinizing hormone and oestrogen. Human Reproduction.

[CR66] Mas M, Alonso C, Hernandez P, Fernandez M, Gutierrez P, Salido E, Baez D (2009). Androgen receptor CAG and GGN polymorphisms and 2D:4D finger ratio in male to female transsexuals. Journal of Sexual Medicine.

[CR67] Mattila AK, Fagerholm R, Santtila P, Miettinen PJ, Taskinen S (2012). Gender identity and gender role orientation in female assigned patients with disorders of sex development. Journal of Urology.

[CR68] Mazur T (2005). Gender dysphoria and gender change in androgen insensitivity or micropenis. Archives of Sexual Behavior.

[CR69] McCarthy MM, Arnold AP (2011). Reframing sexual differentiation of the brain. Nature Neuroscience.

[CR70] McFadden D, Loehlin JC, Breedlove SM, Lippa RA, Manning JT, Rahman Q (2005). A reanalysis of five studies on sexual orientation and the relative length of the 2nd and 4th fingers (the 2D: 4D ratio). Archives of Sexual Behavior.

[CR71] McIntyre MH, Cohn BA, Ellison PT (2006). Sex dimorphism in digital formulae of children. American Journal of Physical Anthropology.

[CR72] McIntyre MH, Ellison PT, Lieberman DE, Demerath E, Towne B (2005). The development of sex differences in digital formula from infancy in the Fels Longitudinal Study. Proceedings of the Royal Society of London B: Biological Sciences.

[CR73] Meyer-Bahlburg HFL (2005). Gender identity outcome in female-raised 46, XY persons with penile agenesis, cloacal exstrophy of the bladder, or penile ablation. Archives of Sexual Behavior.

[CR74] Meyer-Bahlburg HFL (2009). Variants of gender differentiation in somatic disorders of sex development: Recommendations for Version 7 of the World Professional Association for Transgender Health’s Standards of Care. International Journal of Transgenderism.

[CR75] Meyer-Bahlburg HFL (2013). Sex steroids and variants of gender identity. Endocrinology and Metabolism Clinics.

[CR76] Meyer-Bahlburg HFL, Dolezal C, Baker SW, New MI (2008). Sexual orientation in women with classical or non-classical congenital adrenal hyperplasia as a function of degree of prenatal androgen excess. Archives of Sexual Behavior.

[CR77] Mitsui T, Araki A, Miyashita C, Ito S, Ikeno T, Sasaki S, Kitta T, Moriya K, Cho K, Morioka K (2016). The relationship between the second-to-fourth digit ratio and behavioral sexual dimorphism in school-aged children. PLoS ONE.

[CR78] Money J, Schwartz M (1976). Fetal androgens in the early treated adrenogenital syndrome of 46 XX hermaphroditism: Influence on assertive and aggressive types of behavior. Aggressive Behavior.

[CR79] Motta-Mena NV, Puts DA (2017). Endocrinology of human female sexuality, mating, and reproductive behavior. Hormones and Behavior.

[CR80] Munafò MR, Nosek BA, Bishop DV, Button KS, Chambers CD, du Sert NP, Simonsohn U, Wagenmakers E-J, Ware JJ, Ioannidis JP (2017). A manifesto for reproducible science. Nature Human Behaviour.

[CR81] Open Science Collaboration (2015). Estimating the reproducibility of psychological science. Science.

[CR82] Pasterski V, Hindmarsh P, Geffner M, Brook C, Brain C, Hines M (2007). Increased aggression and activity level in 3-to 11-year-old girls with congenital adrenal hyperplasia (CAH). Hormones and Behavior.

[CR83] Polderman TJ, Kreukels BPC, Irwig MS, Beach L, Chan Y-M, Derks EM, Esteva I, Ehrenfeld J, Heijer MD, Posthuma D (2018). The biological contributions to gender identity and gender diversity: Bringing data to the table. Behavior Genetics.

[CR84] Reyes F, Winter J, Faiman C (1973). Studies on human sexual development. I. Fetal gonadal and adrenal sex steroids. Journal of Clinical Endocrinology and Metabolism.

[CR85] Rivas M, Moreira L, Santo L, Marques A, El-Hani C, Toralles M (2014). New studies of second and fourth digit ratio as a morphogenetic trait in subjects with congenital adrenal hyperplasia. American Journal of Human Biology.

[CR86] Schmidt FL, Oh IS, Hayes TL (2009). Fixed-versus random-effects models in meta-analysis: Model properties and an empirical comparison of differences in results. British Journal of Mathematical and Statistical Psychology.

[CR87] Schneider HJ, Pickel J, Stalla GK (2006). Typical female 2nd–4th finger length (2D:4D) ratios in male-to-female transsexuals—Possible implications for prenatal androgen exposure. Psychoneuroendocrinology.

[CR88] Schulz KM, Molenda-Figueira HA, Sisk CL (2009). Back to the future: The organizational–activational hypothesis adapted to puberty and adolescence. Hormones and Behavior.

[CR89] Shrout PE, Rodgers JL (2018). Psychology, science, and knowledge construction: Broadening perspectives from the replication crisis. Annual Review of Psychology.

[CR90] Slijper FM (1984). Androgens and gender role behaviour in girls with congenital adrenal hyperplasia (CAH). Progress in Brain Research.

[CR91] Sterling TD (1959). Publication decisions and their possible effects on inferences drawn from tests of significance-or vice versa. Journal of the American Statistical Association.

[CR92] Trivers R, Manning J, Jacobson A (2006). A longitudinal study of digit ratio (2D:4D) and other finger ratios in Jamaican children. Hormones and Behavior.

[CR93] Turanovic JJ, Pratt TC, Piquero AR (2017). Exposure to fetal testosterone, aggression, and violent behavior: A meta-analysis of the 2D:4D digit ratio. Aggression and Violent Behavior.

[CR94] van Hemmen, J., Cohen-Kettenis, P. T., Steensma, T. D., Veltman, D. J., & Bakker, J. (2017). Do sex differences in CEOAEs and 2D:4D ratios reflect androgen exposure? A study in women with complete androgen insensitivity syndrome. *Biology of Sex Differences, 8*. 10.1186/s13293-017-0132-z. 10.1186/s13293-017-0132-z PMC5389183 28413602

[CR95] Veale JF, Clarke DE, Lomax TC (2010). Biological and psychosocial correlates of adult gender-variant identities: New findings. Personality and Individual Differences.

[CR96] Viechtbauer W (2010). Conducting meta-analyses in R with the metafor package. Journal of Statistical Software.

[CR97] Voracek M, Reimer B, Ertl C, Dressler SG (2006). Digit ratio (2D:4D), lateral preferences, and performance in fencing. Perceptual and Motor Skills.

[CR98] Vujović S, Popović S, Mrvošević Marojević L, Ivović M, Tančić-Gajić M, Stojanović M, Marina LV, Barać M, Barać B, Kovačević M (2014). Finger length ratios in Serbian transsexuals. The Scientific World Journal.

[CR99] Wallien MS, Zucker KJ, Steensma TD, Cohen-Kettenis PT (2008). 2D:4D finger-length ratios in children and adults with gender identity disorder. Hormones and Behavior.

[CR100] Williams TJ, Pepitone ME, Christensen SE, Cooke BM, Huberman AD, Breedlove NJ, Breedlove TJ, Jordan CL, Breedlove SM (2000). Finger-length ratios and sexual orientation. Nature.

[CR101] Wong WI, Hines M (2016). Interpreting digit ratio (2D:4D)–behavior correlations: 2D:4D sex difference, stability, and behavioral correlates and their replicability in young children. Hormones and Behavior.

[CR102] Zucker KJ, Bradley SJ, Oliver G, Blake J, Fleming S, Hood J (1996). Psychosexual development of women with congenital adrenal hyperplasia. Hormones and Behavior.

